# Mechanistic Insights into the Fracture Toughness Enhancement of Nano-TiO_2_ and Basalt Fiber Bar Reinforced Magnesium Phosphate Cement

**DOI:** 10.3390/nano15151183

**Published:** 2025-08-01

**Authors:** Wei-Kang Li, Sheng-Ai Cui, Yu-Peng Li, Ya-Lei Zeng, Guang Zeng, Wei Xia

**Affiliations:** School of Civil Engineering, Southwest Jiaotong University, Chengdu 610031, China; weikang_li@my.swjtu.edu.cn (W.-K.L.); 15841177179@my.swjtu.edu.cn (Y.-P.L.); yalei_zeng@my.swjtu.eu.cn (Y.-L.Z.); 13510498678@my.swjtu.edu.cn (G.Z.); xiawei@my.swjtu.edu.cn (W.X.)

**Keywords:** magnesium phosphate cement, nano-TiO_2_, basalt fiber bar, extended finite element theory, composite material theory, fracture toughness

## Abstract

Magnesium phosphate cement (MPC) exhibits brittleness when utilized as a repair material for bridge decks. To address this issue, this study employs nano-TiO_2_ (NT) and a novel material (basalt fiber bar) as modifiers. A double-K fracture model is developed for the modified MPC to quantitatively evaluate the enhancement of fracture toughness induced by NT and basalt fiber bars. The cracking behavior and toughening mechanisms of the NT and basalt fiber bar reinforced MPC are investigated using extended finite element theory and composite material theory. Additionally, a formula is proposed to calculate the incremental fracture toughness of NT and basalt fiber bar reinforced MPC. The results indicated that NT and basalt fiber bar can effectively enhance the ultimate bending capacity of MPC. The improvement increases with the fiber volume fraction, and noticeable bending hardening occurs when the fiber content exceeds 2%. With the same fiber volume fraction, the peak load can be increased by up to 11.7% with the addition of NT. The crack initiation toughness of the NT group without basalt fiber bars is 58% higher than that of the CC group. The content and diameter of basalt fiber bar are critical parameters affecting the toughness of the NT and basalt fiber bar reinforced MPC.

## 1. Introduction

With rapid economic development, high-speed railways have become the preferred mode of transportation due to their convenience and high passenger capacity [1,2]. Bridges are widely used in high-speed rail construction because they can span complex terrains, minimize land use, and address foundation deformation. The strategy of “bridging instead of road construction” has become essential, especially with recent advancements in high-speed rail technology [3]. However, due to the impact of high-speed, high-density train loads, concrete bridge decks often develop surface cracks and potholes as a result of vehicle–bridge interaction. This leads to the frequent need for repair materials. To ensure proper maintenance of railway infrastructure, including tracks, communication signals, and contact networks, repair materials must exhibit rapid strengthening properties. Additionally, since some high-speed rail lines pass through cold regions such as permafrost areas, these materials must demonstrate high early strength, rapid strength development at low temperatures, and strong adhesion.

Magnesium phosphate cement (MPC) is a promising material for high-speed railway repairs due to its high early strength, rapid strength development at low temperatures, and strong adhesion [4]. However, MPC contains a high proportion of crystalline phases, which give it certain ceramic properties, leading to brittleness and poor deformability. This brittleness can cause cracking under high-impact or bending loads, such as those encountered on pavements, bridge decks, and airport runways [5]. As a result, the application of MPC in repairing high-speed railway bridge decks is limited. Several studies have explored ways to reduce the brittleness of MPC. Ezeldin et al. [6] found that incorporating hooked-end steel fibers improves the bending strength and toughness of MPC. Wagh et al. [7] observed that adding 1% to 3% glass fibers enhances the bending strength and toughness of MPC composites, with a maximum improvement approaching 100%. However, steel and glass fibers are expensive. In contrast, basalt fibers offer advantages such as lower cost, environmental friendliness, high elastic modulus, and strong corrosion resistance. As a result, research has increasingly focused on using basalt fibers to reinforce MPC. Qin et al. [8] found that basalt fibers improve the mechanical properties of MPC mortar, demonstrating higher bending strength and toughness compared to glass fibers at the same fiber volume. Jongsung Sim et al. [9] studied the effects of basalt, steel, and carbon fibers on concrete’s mechanical and durability properties, showing that basalt fibers retain their properties at temperatures up to 600 °C. Qin Yong Ma [10] reported that adding small amounts of nano-SiO_2_ to basalt fiber-reinforced concrete improves both its density and mechanical properties. Feng et al. [11] investigated the synergistic effects of nano-Al_2_O_3_ and steel fibers on the mechanical properties of MPC, finding that these combinations enhance its toughness.

**Figure 1:**
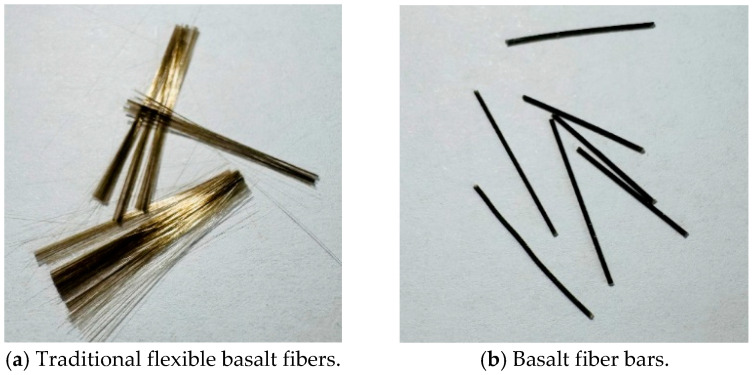
Basalt fibers.

However, current studies primarily focus on modifying the fracture properties of MPC using either nanomaterials or fibers individually. Research on the synergistic effects of nanomaterials and fibers on the toughness of MPC mainly focuses on steel fibers, with limited studies on combining basalt fibers (which are more cost-effective than steel fibers) with nanomaterials. Furthermore, existing research indicates that adding basalt fibers significantly reduces the flowability of MPC, causing issues such as poor workability, construction difficulties, and molding defects [12]. Therefore, a novel material, basalt fiber bars, was selected for this study. Basalt fiber bars, a new type of fiber material, are produced by impregnating and bundling traditional flexible basalt fiber filaments and then processing them into short-cut forms. They offer better dispersion and improved toughness compared to traditional flexible basalt fibers. The basalt fiber morphology is shown in Figure 1 [12,13,14,15]. Research on the synergistic effects of nanomaterials and basalt fiber bars on the fracture toughness of MPC is still lacking.

Thus, this research aims to bridge the gap by analyzing the synergistic effects of NT and basalt fiber bars on the toughness of MPC. NT and basalt fiber bars were selected as modifiers, and the fracture properties of the modified MPC were calculated using the double-K fracture model. The cracking behavior and toughening mechanisms of NT and basalt fiber bar reinforced MPC are investigated using extended finite element theory (XFEM) and composite material theory. Additionally, a formula is proposed to calculate the incremental fracture toughness of NT and basalt fiber bar reinforced MPC.

## 2. Preparation and Experimental Test Design

### 2.1. Materials

**Figure 2:**
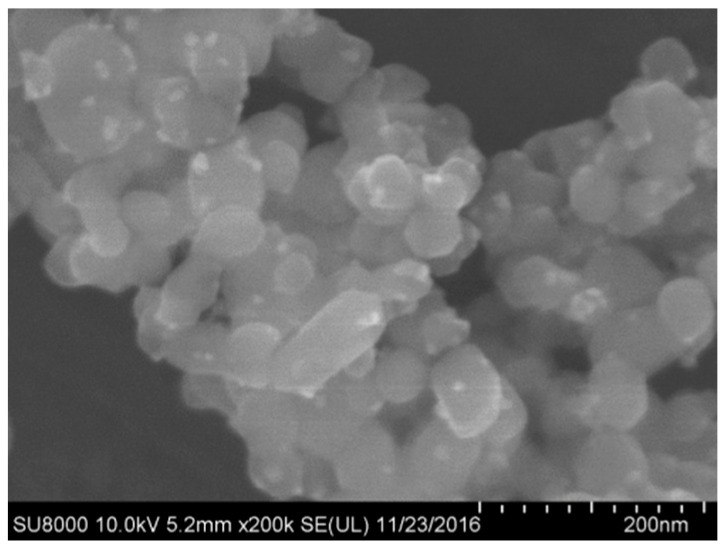
SEM image of nano titanium dioxide.

In this study, calcined MgO (denoted as M) with a purity greater than 90% was used. Ammonium dihydrogen phosphate (denoted as P), the phosphate component in the MPC binding system, was employed. Sodium borate decahydrate (denoted as B) was used as a retarder. The fine aggregate, consisting of quartz sand with a particle size specification of 20–40 mesh, was employed. Nano-TiO_2_ appeared as a white, loose powder with an anatase crystal phase and a primary particle size of 20 ± 5 nm. Sample images and SEM detection images are shown in Figure 2.

Basalt fiber bars were selected for this study, with SiO_2_ and Al_2_O_3_ compositions having purities of 60% and 17%, respectively. The basalt fiber bars have a diameter of 0.4 mm, a length of 12 mm, a density of 2 g/cm^3^, an elastic modulus of 50 GPa, and a tensile strength of 1500 MPa.

### 2.2. Mix Proportion Design and Preparation

An experimental program was designed to ensure the dispersion of the nanomaterials. Sodium hexametaphosphate was used as the dispersing agent to disperse the NT particles. In the concrete mixing procedure, the dispersing agent was first mixed with water. Then, high-speed mechanical stirring was conducted for 6 minutes to disperse the nanomaterials. Finally, the mixture of nanomaterials and water was mixed with MPC. The plain MPC without NT and basalt fiber bars was used as the control concrete (CC-0). The mix proportions are detailed in Table 1.

### 2.3. Test Methods and Data Acquisition

**Figure 3:**
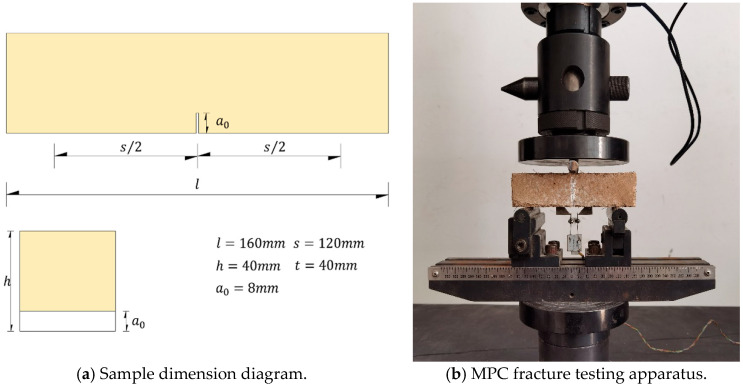
Sample dimension diagram and MPC fracture testing apparatus.

The specimens, with dimensions of 40 mm × 40 mm × 160 mm and notched according to ASTM C1609 [16,17], were prepared for the three-point bending fracture test. The tests were conducted by a universal testing machine, as illustrated in Figure 3. During testing, load (*P*), mid-span deflection (*δ*), and crack opening displacement (*CMOD*) were measured.

## 3. Calculation of Fracture Performance Parameters

### 3.1. Fracture Energy

For notched beam specimens subjected to three-point bending, the fracture energy can be calculated using the formula in Equation (1) [18]:

(1) $$G_{F}=\frac{\left(W_{0}+mg\delta_{max}\right)}{A_{lig}}$$

where $A_{lig}$ is the area of the fracture ligament, calculated as $A_{lig}=t(h-a_{0})$, where *t* is the specimen thickness, *h* is the specimen height, and $a_{0}$ is the initial crack length; $W_{0}$ denotes the work performed by the external load P; m is the mass of the specimen between the two supports, which is 0.45 kg in this study; g is the gravitational acceleration, taken as 9.81 m/s^2^ in this study; and $\delta_{max}$ denotes the maximum deflection at mid-span.

### 3.2. Fracture Toughness

Experimental observations indicate that crack propagation in MPC specimens occurs in three stages: crack initiation, stable growth, and unstable failure. Based on the double-K fracture model for concrete proposed by Xu Shilang et al. [19,20,21,22,23,24,25,26], this study investigates the fracture toughness of MPC specimens. The initiation toughness $K_{Ic}^{ini}$ and the unstable toughness $K_{Ic}^{un}$ are two key control parameters of the double-K fracture model. Cracking begins when the stress intensity factor K reaches $K_{Ic}^{ini}$. When $K_{Ic}^{ini}<K<K_{Ic}^{un}$, the crack grows stably. When $K\geq K_{Ic}^{un},$ the crack experiences unstable propagation.

Xu Shilang et al. [19,20,21,22,23,24,25,26] introduced the linear elastic fracture mechanics (LEFMs) approach to calculate the double-K fracture parameters based on the linear superposition hypothesis. For three-point bending notched beams, the elastic modulus E can be determined using the formula given in Equation (2) [19,20,21,22,23,24,25,26]:

(2) $$E=\frac{1}{tc_{i}}\left[3.70+32.60{tan}^{2}\left(\frac{\pi }{2}\alpha_{h}\right)\right]$$

where $c_{i}$ represents the compliance of the initial linear segment of the P-CMOD curve. The parameter $\alpha_{h}$ is given by $\alpha_{h}=\left(a_{0}+h_{0}\right)/\left(h+h_{0}\right)$, where $h_{0}$ is the thickness of the thin steel plate used for installing the extensometer, which is 5 mm in this experiment.

The formula for calculating the effective crack length is provided in Equation (3) [19,20,21,22,23,24,25,26]:

(3) $$a=\frac{\pi }{2}\left(h+h_{0}\right)arctan\sqrt{\frac{Et}{32.6P}CMOD-0.1135}-h_{0}$$

The critical effective crack length $\alpha_{c}$ can be calculated by substituting the peak load $P_{max}$ and the corresponding crack opening displacement ${CMOD}_{c}$ into Equation (3).

The formula for calculating the stress intensity factor K caused by external loads is given by Equation (4) [19,20,21,22,23,24,25,26]:

(4) $$K_{P}=\frac{1.5\left(P+0.5mg\right)s}{th^{2}}\sqrt{a}F\left(\alpha \right)$$

where $F\left(\alpha \right)$ is defined as $F\left(\alpha \right)=\frac{1.99-\alpha \left(1-\alpha \right)\left(2.15-3.39\alpha +2.7\alpha^{2}\right)}{\left(1+2\alpha \right){\left(1-\alpha \right)}^{3/2}}$, α=a/h. When $P=P_{ini}$ and $a=a_{0}$, the initial crack toughness $K_{Ic}^{ini}$ is obtained. When $P=P_{max}$ and $a=a_{c}$, the critical stability toughness KIcun is determined.

### 3.3. Crack Propagation Resistance

Crack propagation resistance is the ability of a component to resist crack growth under external loads, and it is primarily divided into two stages. Before reaching the crack initiation point, when the main crack has not yet propagated, resistance to crack propagation is mainly provided by the material’s inherent properties. Once the main crack begins to propagate, resistance is primarily provided by cohesive forces [27,28]. The resistance to crack propagation can be represented by Equation (5):

(5) $$K_{R}\left(∆a\right)=K_{Ic}^{ini}+K^{c}(∆a)$$

Here, ∆a denotes the crack growth length, defined as $∆a=a-a_{0}$.

The stress intensity factor resulting from cohesive forces can be calculated using Equations (6) and (7) [29]:

(6) $$K^{c}=\int_{a_{0}}^{a}\frac{2}{\sqrt{\pi a}}\sigma (x)F(u,v)dx$$

(7) $$F\left(u,v\right)=\frac{3.52\left(1-\mu \right)}{{\left(1-v\right)}^{3/2}}-\frac{4.35-5.28\mu }{{\left(1-v\right)}^{1/2}}+\left[\frac{1.3-0.3u^{3/2}}{{\left(1-u^{2}\right)}^{1/2}}+0.83-1.76u\right]\cdot \left[1-(1-u)\right]$$

where u=x/a and v=a/h, where σ(x) is the cohesive force at the crack length x.

**Figure 4:**
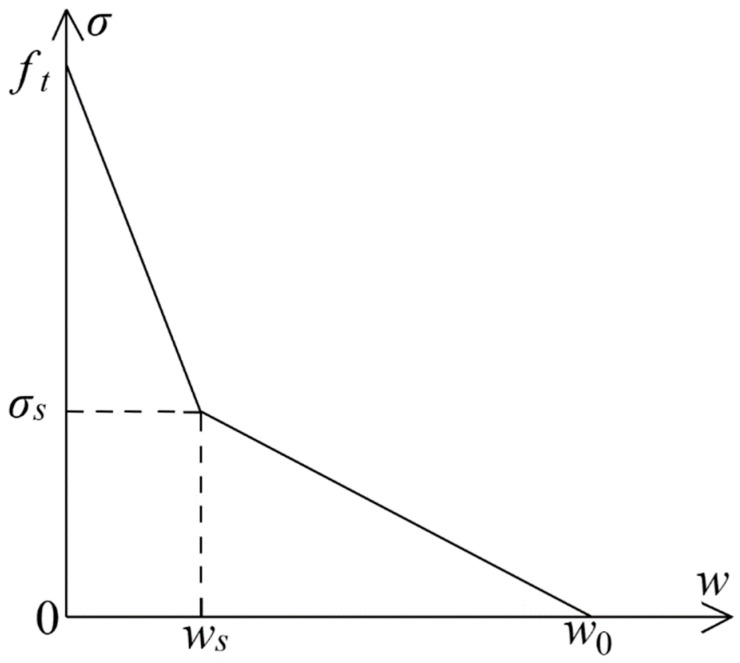
The bilinear softening curve.

The bilinear softening curve used in this study is shown in Figure 4, and its expression is given as follows:

(8) $$\left\{\begin{array}{cc} \sigma =f_{t}-\left(f_{t}-\sigma_{s}\right)w/w_{s}, & 0\leq w\leq w_{s}\\ \sigma =\sigma_{s}\left(w_{0}-w\right)/w_{0}, & w_{s}\leq w\leq w_{0}\\ \sigma =0, & w\geq w_{0} \end{array}\right.$$

where σ represents the cohesive force (MPa); w is the crack opening displacement (mm); $f_{t}$ denotes the tensile strength of the concrete (MPa); $w_{0}$ is the crack opening displacement when σ=0 (mm); $and\;(w_{s},\sigma_{s})$ denote the coordinates of the point of inflection on the softening curve.

Figure 4 illustrates that the shape of the softening curve in the bilinear softening model depends on the values of $w_{s}$, $\sigma_{s}$, and $w_{0}$. The softening constitutive equation proposed by Xu and Reinhardt is utilized in this study [30]:

(9) $$\left\{\begin{array}{l} \sigma_{s}=f_{t}(2-f_{t}w_{s}/G_{F})/\alpha_{F}\\ w_{s}=CTOD_{C}\\ w_{0}=\alpha_{F}G_{F}/f_{t}\\ \alpha_{F}=\lambda -d_{max}/8 \end{array}\right.$$

where $CTOD_{C}$ represents the critical crack tip opening displacement (mm); $d_{max}$ is the maximum aggregate size (mm), which is taken as 0.85 in this study; λ is the correction factor, typically ranging from 5 to 10, and is taken as 8 in this study.

When the crack in the fracture process zone extends to x, the crack opening displacement can be calculated using the following formula [31]:

(10) $$w\left(x\right)=CMOD\sqrt{{\left(1-x/a\right)}^{2}+\left(1.081-1.149a/h\right)[x/a-{\left(x/a\right)}^{2}]}$$

This study applies the theory outlined in the reference, which accounts for the variation in the fracture process zone length with crack propagation; it divides crack propagation resistance into four distinct stages [32]:

(1) When $a=a_{0}$, the specimen has not yet cracked and is in the elastic stage with zero cohesive force.

(11) $$\sigma \left(x\right)=0,\;a=a_{0}$$

**Figure 5:**
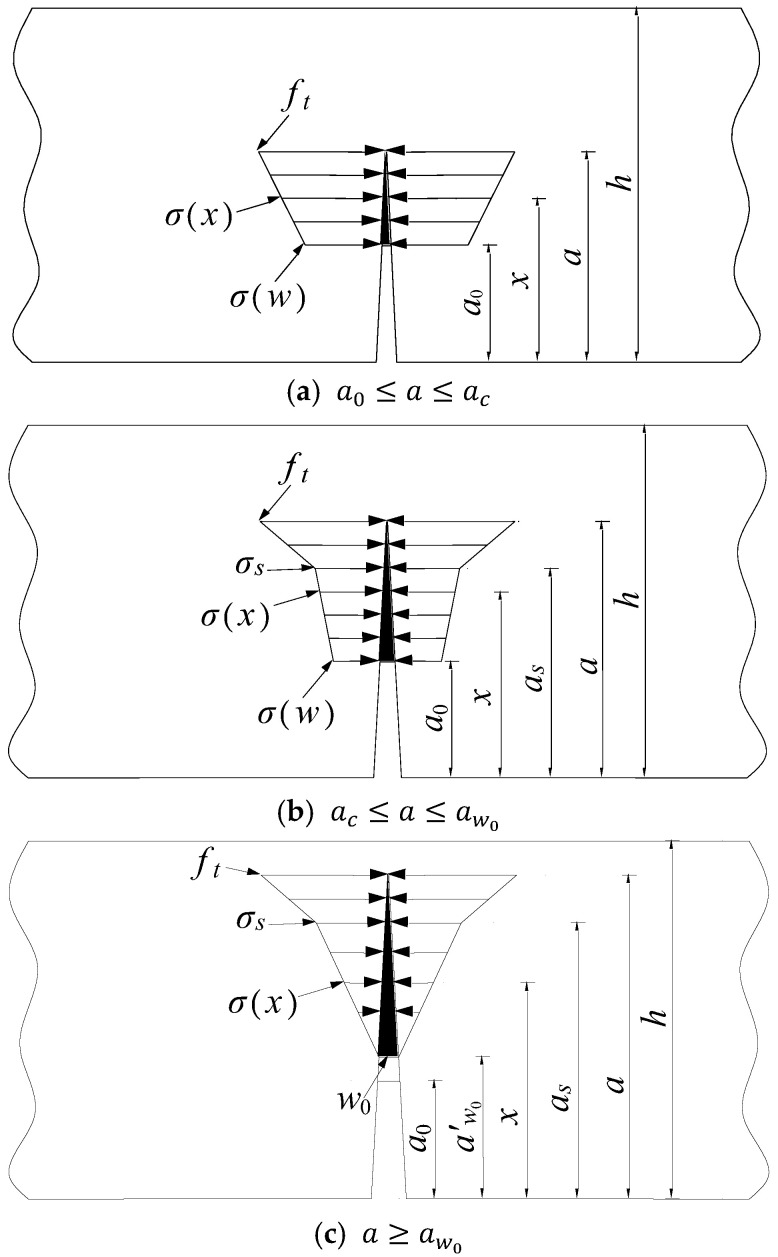
Distribution map of cohesive strength.

(2) When $a_{0}\leq x\leq a_{c}$, the crack begins to propagate stably. Figure 5a illustrates the cohesive force distribution in the fracture process zone, and the cohesive force distribution function is provided by the following:

(12) $$\sigma \left(x\right)=\sigma \left(w\right)+\frac{\left[f_{t}-\sigma \left(w\right)](x-a_{0}\right)}{a-a_{0}},\;\;a_{0}\leq x\leq a_{c}$$

where σ(w) is the initial cohesive force at the crack tip.

(3) When $a_{c}\leq a\leq a_{w_{0}}$, Figure 5b displays the distribution of cohesive forces. The function describing this distribution is as follows:

(13) $$\sigma \left(x\right)=\left\{\begin{array}{ll} \sigma_{1}\left(x\right)=\sigma \left(w\right)+\frac{\left[\sigma_{s}-\sigma \left(w\right)\right]\left(x-a_{0}\right)}{a_{s}-a_{0}}, & a_{0}\leq x\leq a_{s}\\ \sigma_{2}\left(x\right)=\sigma_{s}+\frac{\left(f_{t}-\sigma_{s}\right)\left(x-a_{s}\right)}{a-a_{s}}, & a_{s}\leq x\leq a \end{array}\right.$$

where $a_{s}$ is the inflection point of the cohesive force. Substitute $w\left(x\right)=w_{s}$ into Equation (10) to solve for $x=a_{s}$.

(4) When $\geq a_{w_{0}}$, the cohesive force distribution is shown in Figure 5c. The cohesive force distribution function is as follows:

(14) $$\sigma \left(x\right)=\left\{\begin{array}{cc} \sigma_{1}\left(x\right)=0, & a_{0}\leq x\leq a_{w_{0}}^{\prime }\\ \sigma_{2}\left(x\right)=\frac{\sigma_{s}\left(x-a_{w_{0}}^{\prime }\right)}{a_{s}-a_{w_{0}}^{\prime }}, & a_{w_{0}}^{\prime }\leq x\leq a_{s}\\ \sigma_{2}\left(x\right)=\sigma_{s}+\frac{\left(f_{t}-\sigma_{s}\right)\left(x-a_{s}\right)}{a-a_{s}}, & a_{s}\leq x\leq a \end{array}\right.$$

where $w\left(x\right)=w_{0}$ yields $x=a_{w_{0}}^{\prime }$ in Equation (10).

At this point, the length of the fracture process zone is $a-a_{w_{0}}^{\prime }$, and the length of the fracture process zone in the previous three stages is $a-a_{0}$.

## 4. Numerical Simulation of MPC Fracture Testing Based on XFEM

The Extended Finite Element Method (XFEM) [33,34,35,36], based on the fundamental concept of unit decomposition, introduces additional functions into the conventional finite element displacement mode to account for the discontinuity of crack surfaces and the singularity at the crack tip. This method does not require the crack surface to align with the element edges, overcoming the limitations of conventional finite element analysis, such as complex mesh generation and the need for remeshing after crack propagation.

In this study, key constitutive parameters were determined through experiments. A discrete modeling approach was adopted, considering the interaction between fibers and MPC using an embedded method [37]. Numerical simulations of fracture tests for NT groups with 1% and 2% fiber contents were performed using XFEM.

### 4.1. Extended Finite Element Method

XFEM was used in this study, with the theory provided in Appendix A.

### 4.2. Constitutive Model Parameters Test

#### 4.2.1. MPC Mortar

**Figure 6:**
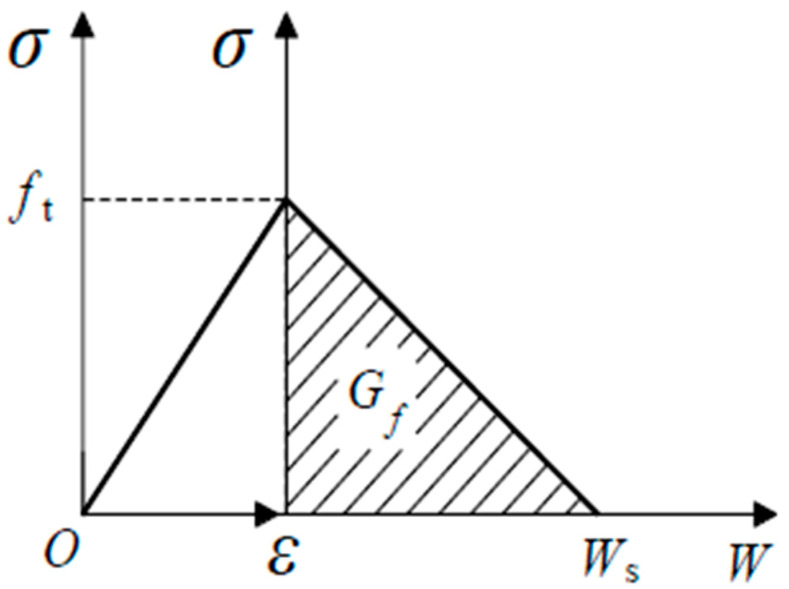
MPC mortar constitutive relationship diagram.

In this study, the NT group is treated as a homogeneous solid. The elastic modulus, as determined experimentally, is 30 GPa. The constitutive relationship is modeled using a simplified bilinear law. It is assumed that the nano-enhanced MPC behaves as an elastic material before reaching the peak stress. After reaching the peak stress, the material’s softening behavior is characterized by stress versus crack opening displacement. A schematic representation of the mortar’s constitutive relationship is shown in Figure 6.

In the figure, $f_{t}$ represents the tensile strength of the material; $G_{f}$ is the fracture energy, which is taken as 0.114 N/mm based on the previously calculated results; W denotes the opening displacement; and $W_{S}$ represents the ultimate opening displacement.

**Figure 7:**
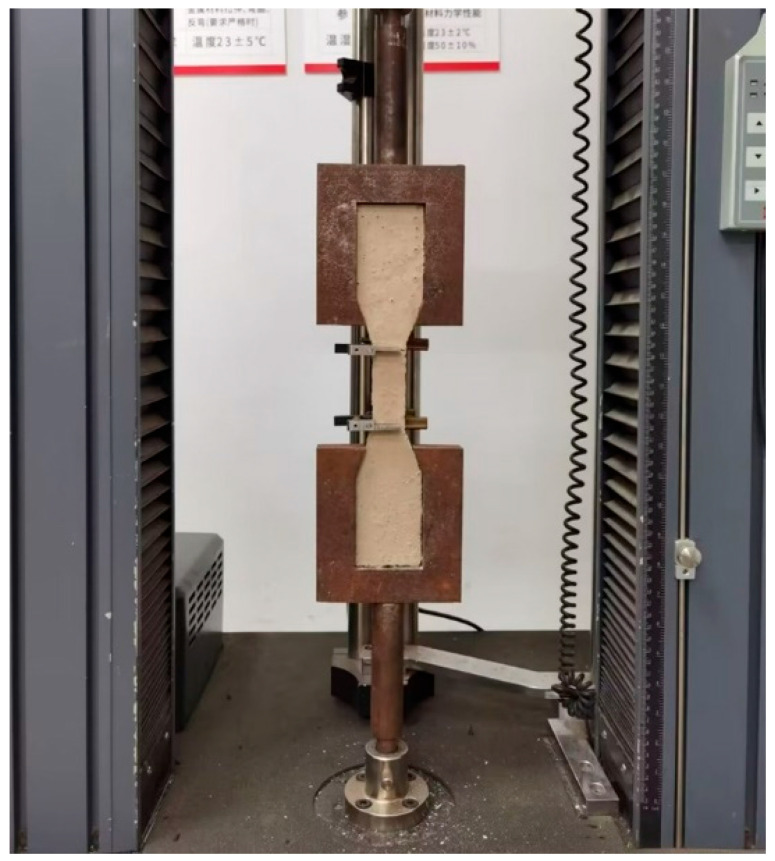
MPC uniaxial tensile test.

In the extended finite element model, the failure criteria include the maximum principal stress and strain criteria, the maximum nominal principal stress and strain criteria, and the quadratic nominal stress and strain criteria. Among these, only the maximum principal stress and strain criteria allow for the free propagation of cracks, with the propagation direction being orthogonal to the maximum principal stress. Therefore, this study selects the maximum principal stress criterion as the cracking criterion for the model. In the initial phase, the mesoscale elements are treated as elastic materials, with their mechanical properties represented by the elastic modulus and Poisson’s ratio. As the stress on the element increases, when the maximum principal tensile stress reaches its ultimate tensile stress, the element begins to undergo tensile damage. To determine the ultimate tensile stress of the nano-enhanced MPC matrix, a uniaxial tensile test was designed, as shown in Figure 7. The final mortar constitutive parameters are listed in Table 2.

#### 4.2.2. Basalt Fiber Bar Constitutive Relationship

Since the model uses an embedded approach to represent the interaction between the mortar and fibers, the fiber pull-out effect cannot be considered, which impacts the accuracy of the model. To more accurately replicate the process of fibers being pulled and extracted from the mortar matrix, a pull-out test for MPC fibers was designed to determine the fiber constitutive relationship.

**Figure 8:**
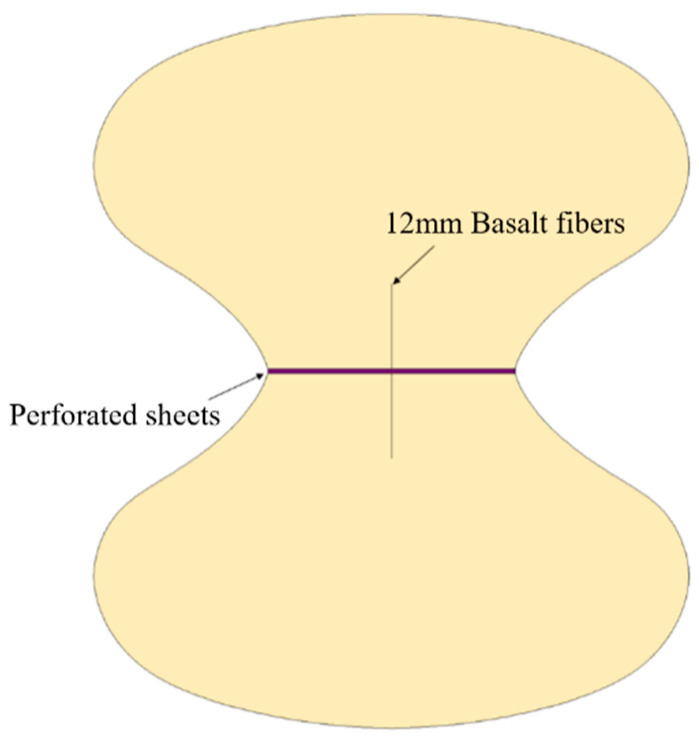
Eight-shaped fiber pull-out MPC specimens.

The fiber pull-out specimens were molded using a figure-eight mold, with the mortar cast in two layers: upper and lower. A thin plate with holes was placed in the middle and coated with a release agent. The fibers were inserted into the holes, ensuring equal distances from the top and bottom ends. The mixture was uniformly compacted. A schematic diagram of the specimen is shown in Figure 8.

**Figure 9:**
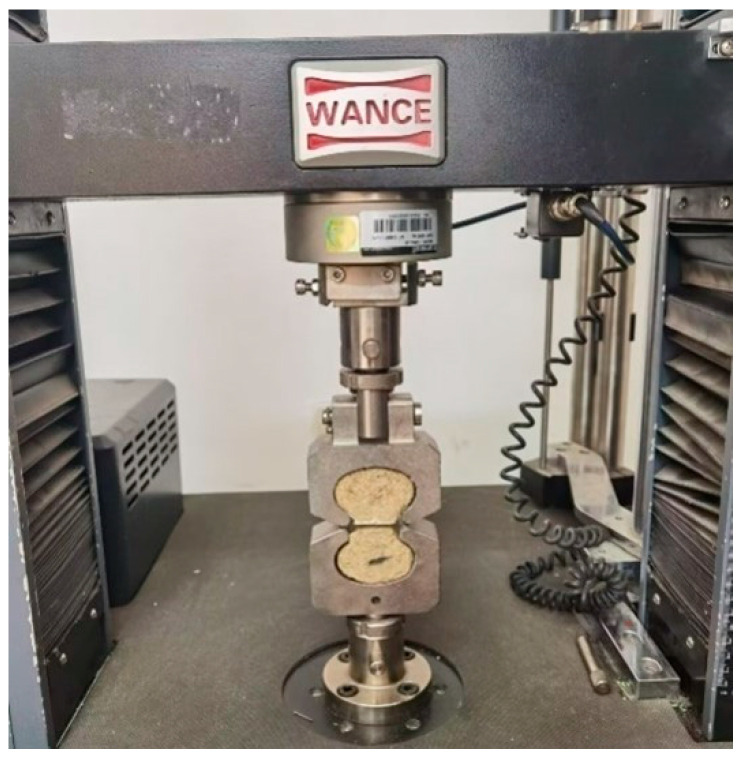
Fiber pull-out test.

**Figure 10:**
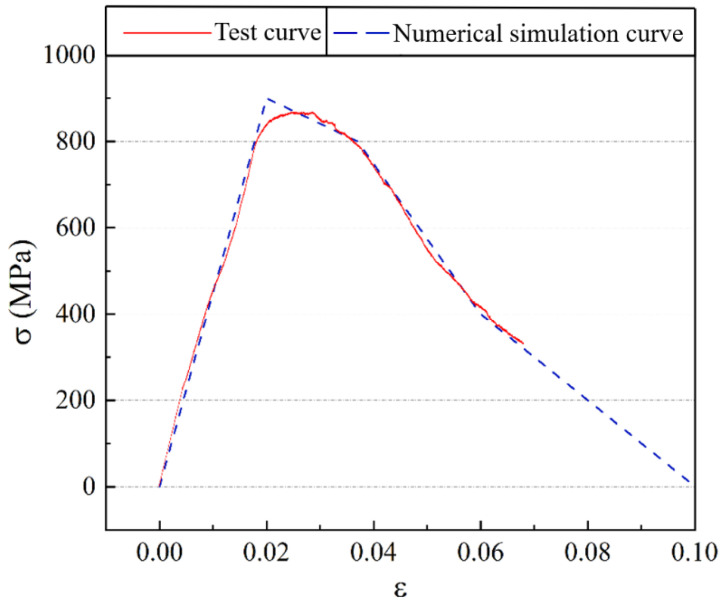
Fiber constitutive curve.

After curing for 1 day, a figure-eight specimen tensile test was conducted using an electro-hydraulic servo universal testing machine with a 2 kN load cell. The testing setup is shown in Figure 9. The measured load-displacement curve was converted into an equivalent stress–strain curve for the fibers, as shown by the red line in Figure 10. In Abaqus 2017, the basalt fiber bar plastic constitutive curve was input in tabular form, as shown by the dashed line in Figure 10.

### 4.3. Random Fiber Generation

The random fiber generation method used in this model is based on the Python 3.8 programming language. In the simplest three-dimensional numerical model, it is assumed that all fibers are straight segments with a diameter of 12 mm. Their positions and angles are randomized. The total number of fibers is calculated based on the specimen dimensions and fiber volume fraction. The calculation formula is given by Equation (15):

(15) $$N=\frac{V\cdot \rho_{f}}{\frac{\pi }{4}D^{2}L}$$

where N represents the total number of fibers in a single specimen; V is the volume of the specimen; $\rho_{f}$ denotes the fiber density; D is the fiber diameter; and L is the fiber length.

**Figure 11:**
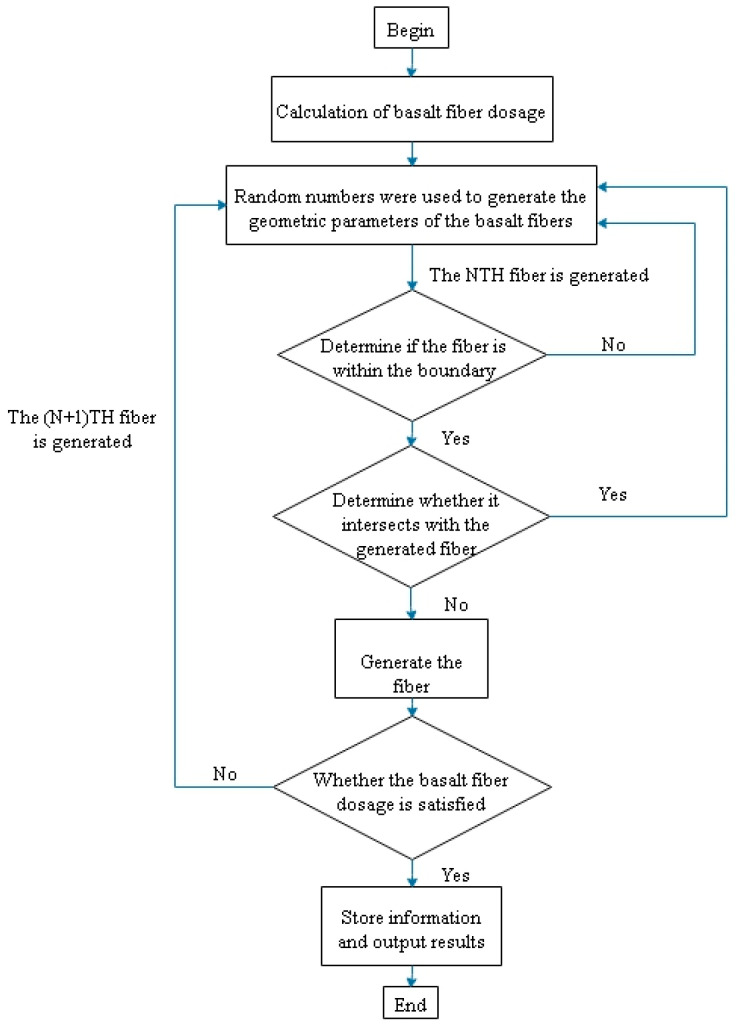
Flowchart of random fiber generation.

**Figure 12:**
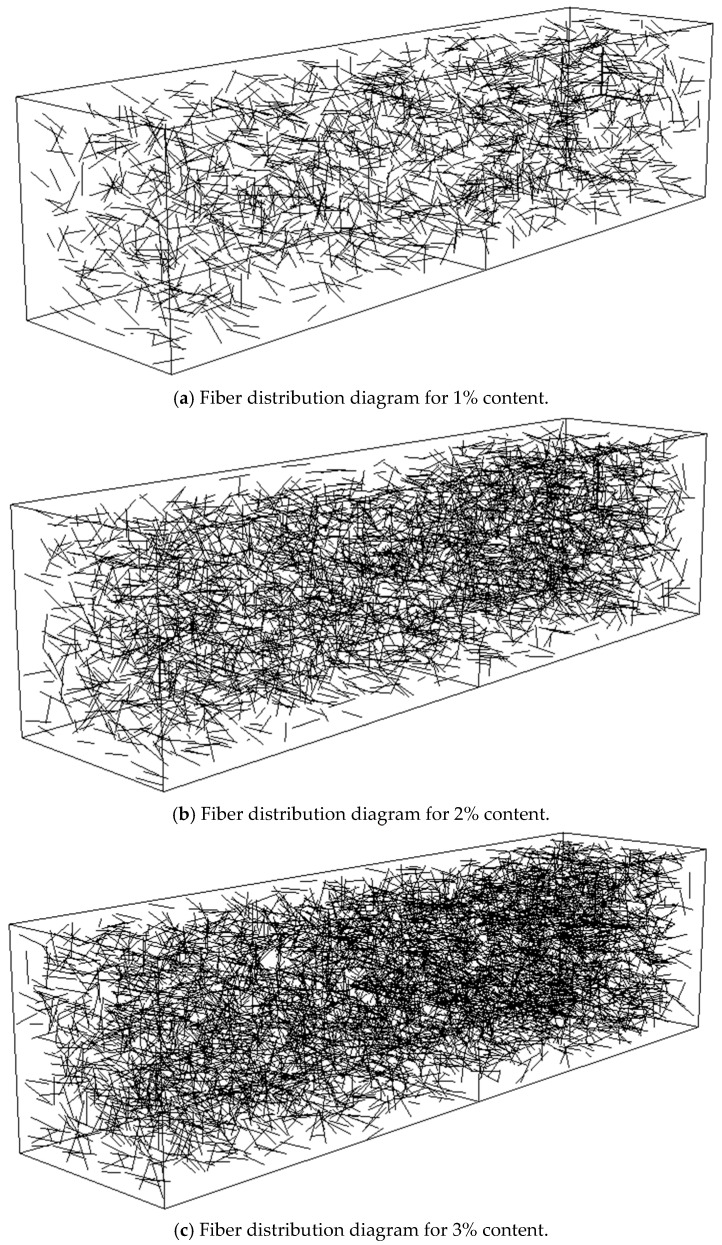
Fiber content distribution diagram.

Using Python’s random number generation, random starting coordinates for fibers, random horizontal angles, and random vertical angles are established. The endpoint coordinates of the fibers are calculated based on the fiber length and direction angles. It is then determined whether both endpoints are within the specimen and whether they intersect. If the conditions are satisfied, the fiber coordinates are output; otherwise, the fiber coordinates are regenerated. The flowchart of the random fiber generation program is shown in Figure 11. Random fiber models with 1%, 2%, and 3% volume fractions are illustrated in Figure 12.

### 4.4. Microscale Fracture Simulation Testing and Analysis of NT and Fiber Reinforced MPC

Based on the previously measured constitutive model and the constructed NT and basalt fiber bar reinforced MPC geometric model, a three-point bending fracture test of pre-cracked MPC specimens (40 mm × 40 mm × 160 mm) is simulated using XFEM, incorporating pre-cracked geometry. The impact of fiber volume fraction on the fracture performance parameters of MPC is evaluated.

**Figure 13:**
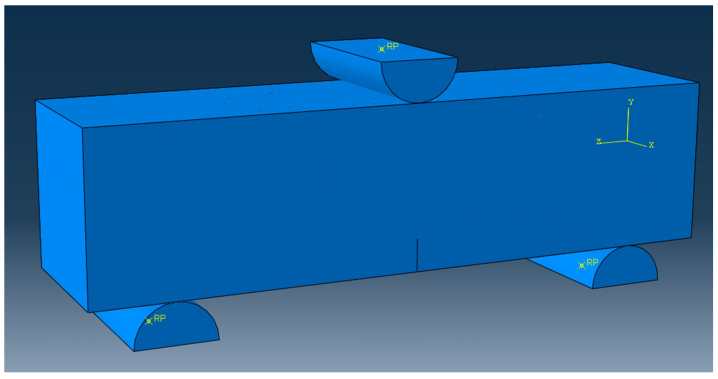
Finite element model of NT and fiber reinforced MPC notched beam.

The model setup is shown in Figure 13, which includes the construction of a semi-circular support and a loading head. Three contact pairs are defined: the lower support is bonded to the bottom surface, and the upper loading fixture is defined as hard contact with the top surface, with a friction coefficient of 0.2. To obtain the full load-displacement curve, a displacement-controlled loading method is used, with a loading displacement of 5 mm in the negative y-direction.

## 5. Analysis of Toughening Mechanisms in NT and Basalt Fiber Bar Reinforced MPC Based on the Composite Material Theory

Based on the above research, it has been found that basalt fiber bars can effectively enhance the fracture performance of MPC. To further reveal the toughening mechanism of basalt fiber bars in MPC from a micromechanical perspective, this section establishes a fracture energy calculation model for NT and basalt fiber bar reinforced MPC based on the composite material theory. The fracture energy under different fiber volume fractions is predicted and calculated.

The calculation basis of the composite material theory assumes that the performance of composite materials equals the sum of the performances of their individual components, multiplied by their respective volume fractions [38]. When the fiber volume fraction in the composite cementitious material does not exceed 2%, the interaction between components can be neglected in the composite material theory analysis. This study aims to reveal the toughening mechanism of basalt fiber bars in MPC from a micromechanical perspective. Therefore, fiber volume fractions of 1% and 2% were selected for calculation in the NT groups.

The application of composite material theory and the establishment of specific parameters in this study are provided in Appendix A.

## 6. Results and Discussion

### 6.1. The Three-Point Bending Fracture Test

**Figure 14:**
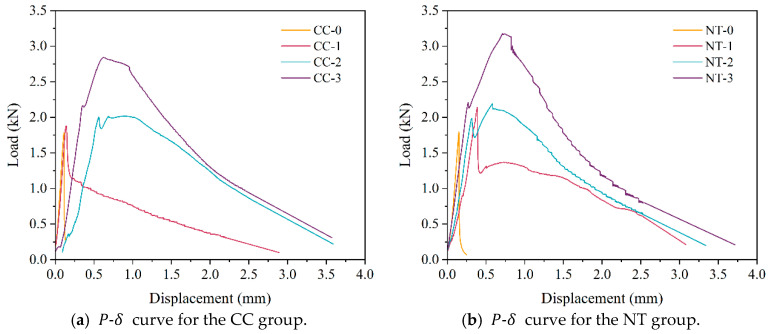
Load-deflection curves.

**Figure 15:**
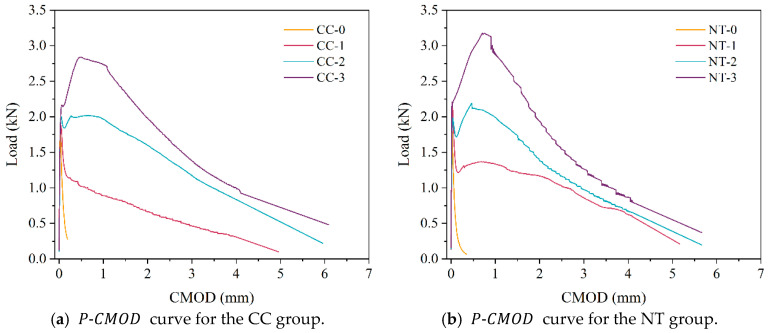
Load-crack opening displacement curves.

The load-deflection (P-δ) curves and load-crack opening displacement (P-CMOD) curves are shown in Figure 14 and Figure 15.

As shown in Figure 14, the maximum load ($P_{max}$) increases with higher fiber content, and the rate of increase also grows with the fiber volume. Due to the crack-bridging effect of modified basalt fibers, the load-displacement curves of modified basalt fiber-reinforced MPC exhibit three distinct stages: linear increase, non-linear rise, and gradual decline. In the initial loading stage, the behavior of modified basalt fiber-reinforced MPC is similar to that of ordinary concrete. The low load results in the crack tip stress not reaching the critical value, and the microcracks within the MPC remain stable. During this phase, cracks do not propagate, and the external load increases linearly with specimen deformation. As the load continues to rise, the crack tip stress reaches the critical value, marking the initiation of cracking, and the curve transitions from linear to non-linear. Once the load reaches its peak value, internal cracks rapidly propagate, and macro-cracks appear on the specimen surface. However, the bridging effect of the fibers effectively impedes further crack propagation. When the fiber content exceeds 2%, a noticeable post-peak hardening phenomenon is observed, indicating that modified basalt fibers significantly enhance the load-bearing capacity and deformation ability of the specimen, thereby improving its toughness. The addition of traditional basalt fibers significantly reduces workability, with a maximum content of 1.5%. At this level, no noticeable post-peak hardening phenomenon can be observed in the traditional basalt fiber-reinforced MPC [8]. Additionally, combining Figure 14a,b reveals that, for the same fiber content, the incorporation of nano-TiO_2_ further increases the peak load of MPC, with a maximum increase of 11.7%. This increase in peak load leads to further improvements in fracture toughness and other fracture performance parameters of the specimen. This result demonstrates that nano-TiO_2_, similar to other nanomaterials, can enhance the toughness of MPC [10,11].

### 6.2. Fracture Energy

**Figure 16:**
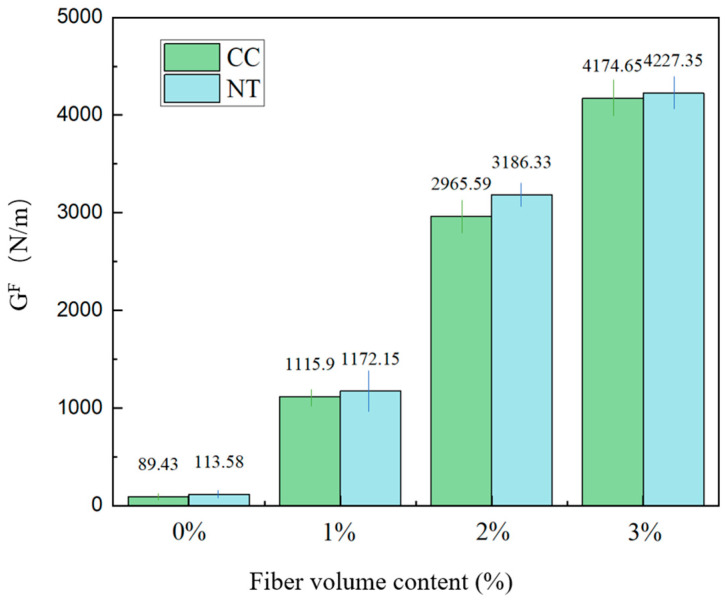
Fracture toughness under different fiber contents.

The fracture energy of MPC, calculated from the experimental P-δ curves, is illustrated in Figure 16.

As shown in Figure 16, the fracture energy for each group in both experimental groups increases significantly with the higher fiber content. Additionally, for the same fiber content, the NT group exhibits higher fracture energy. It can indicate that NT can synergistically interact with basalt fiber bars to further enhance the toughness of the MPC [10].

### 6.3. Fracture Toughness

**Figure 17:**
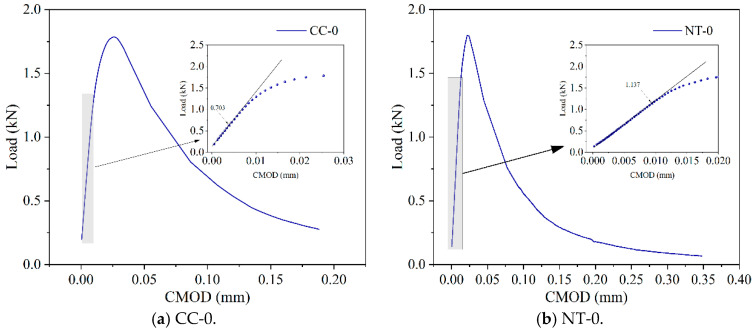

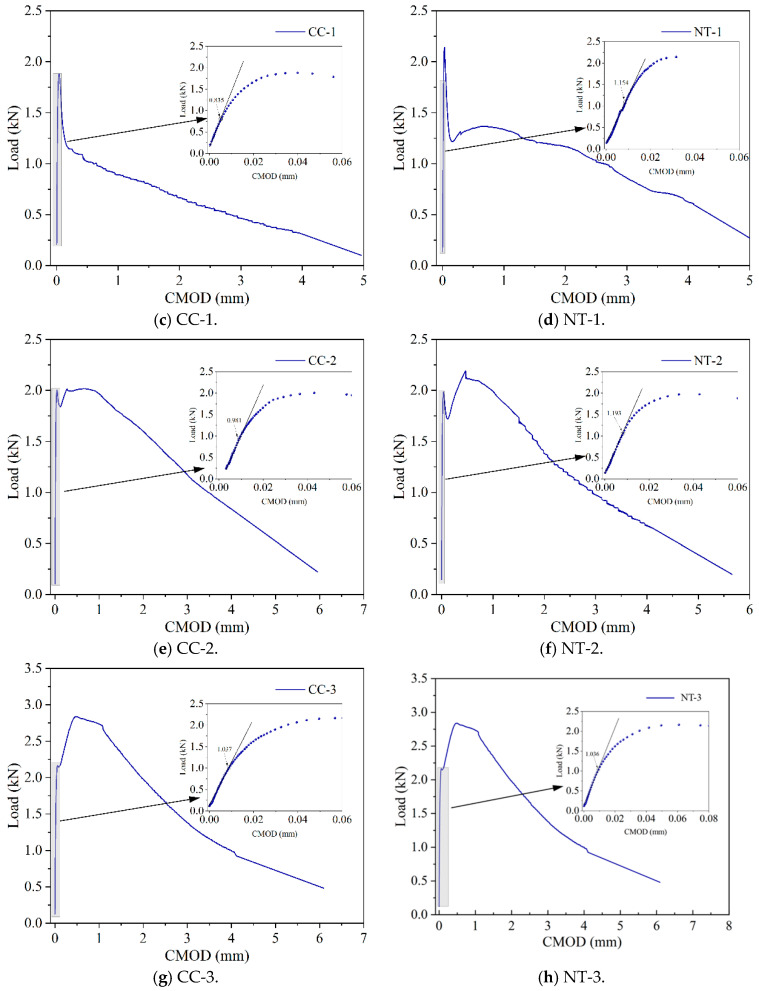
P-CMOD curve.

In this experiment, the value of the load at the transition from linear to non-linear in the P-CMOD curve is selected as the crack initiation load. The crack initiation points for each condition are determined as shown in Figure 17.

**Figure 18:**
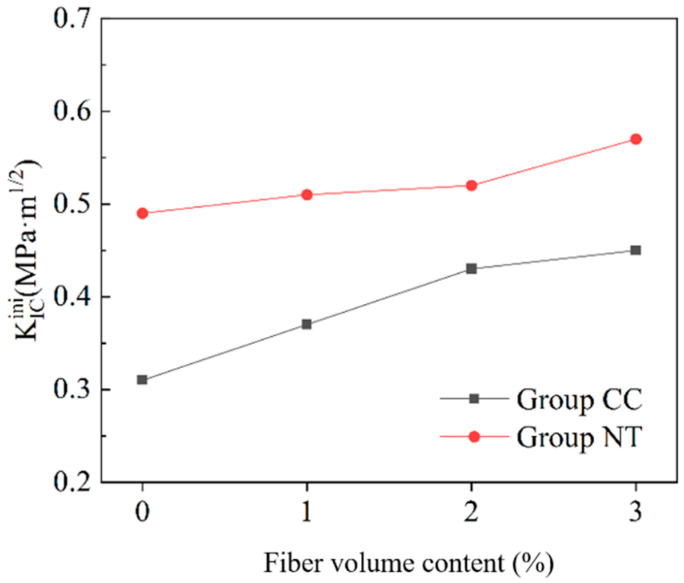
Initial crack toughness $K_{IC}^{ini}$ for different conditions.

**Figure 19:**
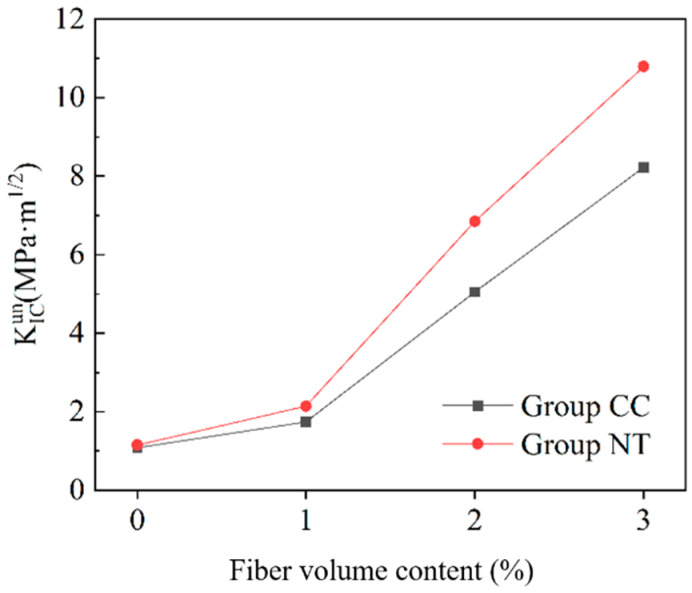
Unstable toughness $K_{IC}^{ini}$ for different conditions.

By combining the crack initiation load with the P-CMOD curve, the crack initiation toughness and critical stability toughness of MPC with different fiber contents are calculated using Equation (4). The results are shown in Figure 18 and Figure 19.

From Figure 18, it can be observed that, at the same fiber content, the crack initiation toughness of the NT group is consistently higher than that of the reference group. As the fiber content increases, the crack initiation toughness of both the CC and NT groups improves to varying extents. When the basalt fiber bars content is 0, the crack initiation toughness of the NT group is 58% higher than that of the CC group. This improvement is attributed to the enhanced strength of the MPC matrix due to the addition of NT, which increases the crack resistance of the MPC specimens. For the reference group, the crack initiation toughness increases by 47% as the fiber content rises from 0% to 3%. For the NT group, the crack initiation toughness increases by 13% under the same conditions. The impact of basalt fiber bars on enhancing crack initiation toughness is more significant when the strength of the MPC matrix is lower.

From Figure 19, it is evident that, at the same fiber content, the critical stability toughness of the NT group is higher than that of the CC group. For the CC group, the critical stability toughness improves by 61%, 368%, and 661% for fiber contents of 1%, 2%, and 3%, respectively, compared to the baseline condition. For the NT group, the critical stability toughness increases by 86%, 496%, and 838% for the same fiber contents, relative to the baseline condition. This indicates that the inclusion of basalt fiber bars significantly enhances the fracture toughness of MPC, with the improvement further amplified by the addition of NT.

### 6.4. Crack Propagation Resistance

**Figure 20:**
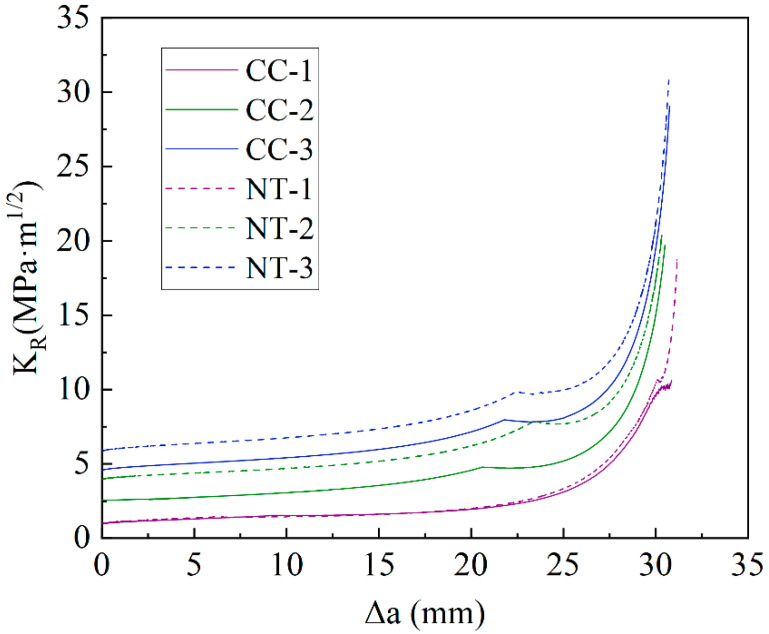
Crack propagation resistance curves for different conditions.

**Figure 21:**
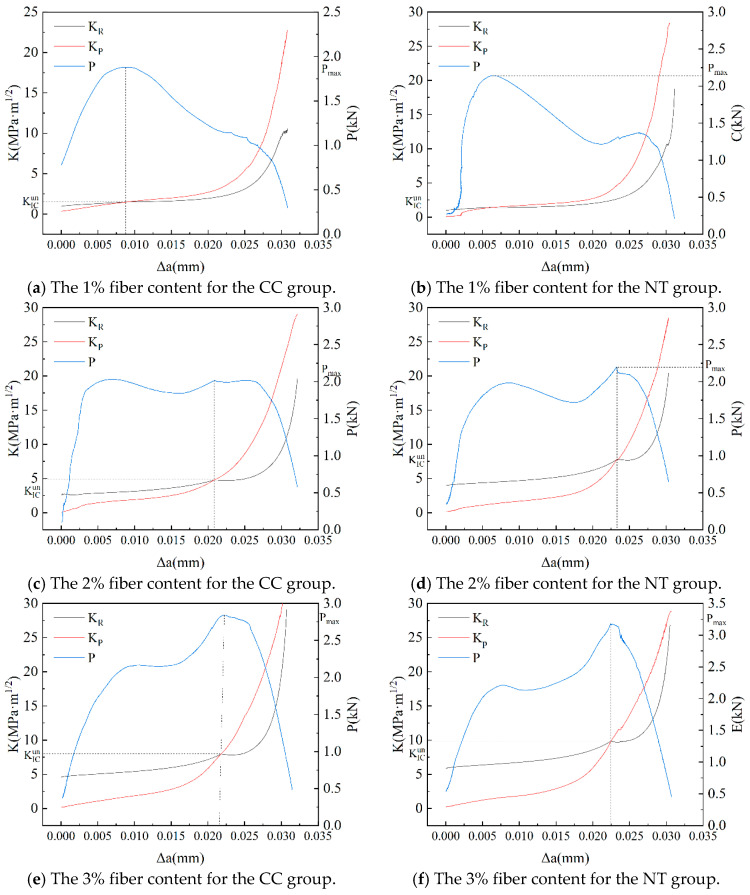
Analysis of crack propagation stability for MPC under different conditions.

The resistance curves $K_{R}$ for the CC and NT groups under various conditions were calculated and summarized in Figure 20. To assist in analyzing the relationship between crack propagation resistance $K_{R}$ and the stress intensity factor $K_{P}$ for different fiber contents, Figure 21 illustrates the correlations among $K_{R}$, $K_{P}$, external load P, and crack propagation length Δa.

From Figure 21, it can be observed that the crack propagation stability of the NT and CC groups follows the same pattern under identical fiber contents. When $\Delta a<a_{c}-a_{0}$, the stress intensity factor $K_{P}$ is lower than the resistance curve $K_{R}$, indicating that the crack in the MPC is in the stable propagation phase. When $\Delta a>a_{c}-a_{0}$, $K_{P}$ exceeds $K_{R}$, indicating that the crack is in the unstable propagation phase. The intersection points of the $K_{P}$ and $K_{R}$ curves correspond to the crack propagation length Δa at the peak load, where $K_{P}=K_{R}=K_{IC}^{un}$. Additionally, when the fiber content is greater than 2%, the peak load occurs after the first load peak, leading to a significant increase in $CMOD_{C}$ and a corresponding increase in the critical stability toughness $K_{IC}^{un}$. Figure 20 shows that, at the same fiber content, the crack propagation resistance of the NT group is higher than that of the CC group, indicating that the addition of NT effectively enhances the crack propagation resistance of MPC.

### 6.5. Microscale Fracture Simulation Testing and Analysis of NT and Fiber Reinforced MPC

**Figure 22:**
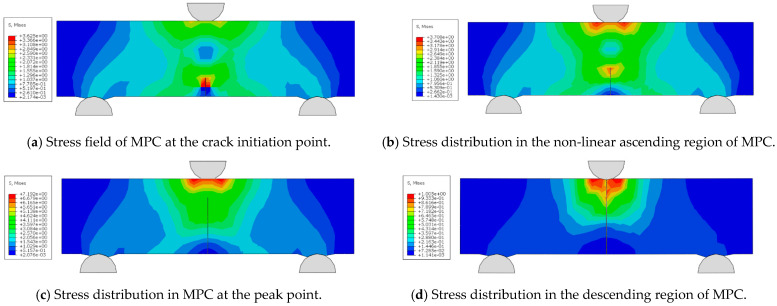
Stress distribution of MPC at different stages.

The MPC mortar is discretized using C3D8R elements (three-dimensional stress hexahedral mesh), while the fibers are represented by T3D2 truss elements. After assigning material properties to the elements, the model is analyzed using the ABAQUS/Standard implicit solver. The Mises stress contour plots for the mortar at different stages are extracted and shown in Figure 22.

**Figure 23:**
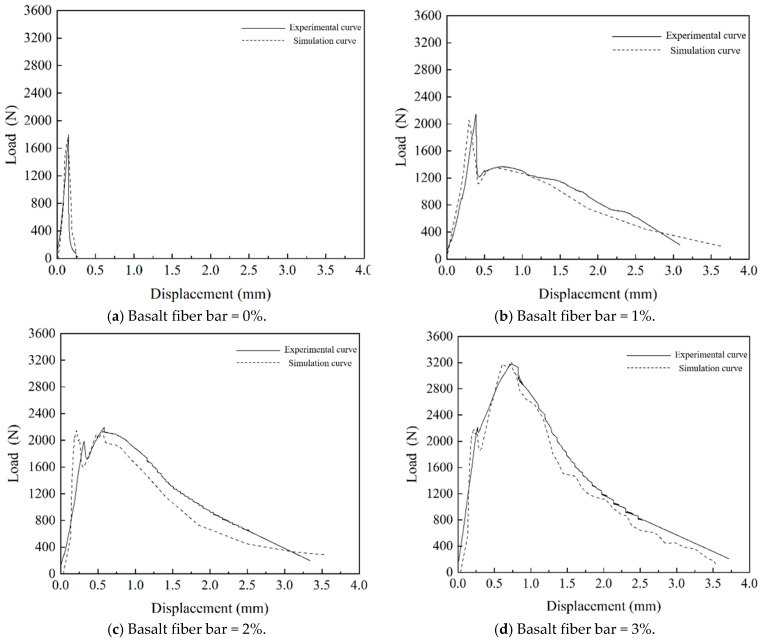
Load-displacement simulation curves for nano-enhanced MPC with different fiber contents.

From Figure 22, it is evident that substantial stress concentration develops at the crack tip during the initial loading phase. When the concrete at the crack tip reaches the maximum principal stress, the crack begins to propagate, causing stress release on the crack surface. As the crack continues to grow, stress concentration reoccurs at the crack tip. This process of stress concentration, cracking, and release repeats continuously until the specimen is completely destroyed. The load-displacement curves for different groups and their comparison with the fracture test curve are shown in Figure 23.

From Figure 23, it can be observed that the peak strength and peak displacement of the simulated curve closely match those of the experimental curve. The simulated curve fluctuates around the experimental curve. Therefore, the simulation accuracy of the MPC fracture test using the extended finite element method in ABAQUS is relatively high.

### 6.6. Analysis of Toughening Mechanisms in NT and Basalt Fiber Bar Reinforced MPC Based on the Composite Material Theory

For notched beam specimens, the theoretical formula should also account for the influence of the notch-to-depth ratio on the fracture energy calculation. Therefore, the increment in fracture energy due to the incorporation of basalt fiber bars can be expressed by Equation (16):

(16) $$W_{t}=(1-a_{0}/h)N\frac{1}{24}\pi d_{f}\tau \frac{{{(l}_{f}^{crit})}^{3}}{l_{f}}$$

The calculation parameters for each condition are shown in Table 3. From the table, it can be observed that the theoretical and experimental errors in the increment of fracture energy ΔG are both within 10%, indicating a good agreement with the experimental results.

## 7. Conclusions, Challenges, and Future Prospects

This study investigates the effects of varying amounts of basalt fiber bars and NT on the fracture performance of MPC from both fracture mechanics and micromechanics perspectives. The toughening mechanism of NT and basalt fiber bars in MPC is explored. Additionally, finite element simulations and theoretical calculations of fracture energy for modified basalt fiber-reinforced MPC materials are performed based on extended finite element theory and composite material theory. The following conclusions are drawn:

(1) Basalt fiber bars can significantly enhance the ultimate bending capacity of MPC notched beams. The improvement increases with the fiber volume fraction, and noticeable bending hardening occurs when the fiber content exceeds 2%.
(2) NT also enhances the ultimate load-carrying capacity of MPC notched beams. For the same fiber volume fraction, the peak load can be increased by up to 11.7% with the addition of NT.
(3) The crack propagation resistance K_R_ of MPC shows a positive correlation with the crack propagation length and exhibits a trend of slow initial growth followed by accelerated growth. Furthermore, for the same fiber volume fraction, the NT group shows higher crack propagation resistance compared to the CC group, indicating that NT has a certain effect on improving the crack propagation resistance of MPC.
(4) The formula for calculating the increment in fracture energy of MPC cementitious materials due to basalt fibers, established using composite material theory, aligns closely with experimental data, with an error within 10%.
(5) The NT and basalt fiber bar reinforced MPC undergoes repeated cycles of stress concentration, cracking, and release during the fracture process until failure occurs. In contrast, this behavior is absent in the MPC without basalt fiber bars.
(6) The content and diameter of basalt fiber bars are critical parameters affecting the toughness of NT and basalt fiber reinforced MPC.

MPC is an expensive rapid repair material, and the addition of basalt fiber bars and nano-TiO_2_ further increases its application cost. If a more cost-effective nanomaterial can be selected as a replacement for nano-TiO_2_, the cost of modified MPC can be reduced, making it more favorable for engineering applications.

## Figures and Tables

**Table 1:** MPC mix proportions (Kg/m^3^).

MIX ID	Binder	Sodium Borate Decahydrate	Quartz Sand	Water	NT	Basalt Fiber Bars
CC-0	750 (MgO) + 250 (NH_4_H_2_PO_4_)	52.5	1000	180	0	0
CC-1	750 (MgO) + 250 (NH_4_H_2_PO_4_)	52.5	1000	180	0	17.4
CC-2	750 (MgO) + 250 (NH_4_H_2_PO_4_)	52.5	1000	180	0	34.8
CC-3	750 (MgO) + 250 (NH_4_H_2_PO_4_)	52.5	1000	180	0	52.2
NT-0	750 (MgO) + 250 (NH_4_H_2_PO_4_)	52.5	1000	180	25	0
NT-1	750 (MgO) + 250 (NH_4_H_2_PO_4_)	52.5	1000	180	25	17.4
NT-2	750 (MgO) + 250 (NH_4_H_2_PO_4_)	52.5	1000	180	25	34.8
NT-3	750 (MgO) + 250 (NH_4_H_2_PO_4_)	52.5	1000	180	25	52.2

**Table 2:** Values for mortar constitutive parameters.

Material	Elastic Modulus (GPa)	Poisson’s Ratio	Tensile Strength (MPa)	Fracture Toughness (N/mm)
Mortar	30	0.2	3.6	0.114

**Table 3:** Calculation parameter results for different conditions.

MIX ID	Primary Calculation Parameters							Error
	$\eta_{0}$	$\eta_{l}$	$\sigma_{fc}^{u}$	$\sigma_{fc}^{b}$	$l_{f}^{crit}$	τ	ΔG	
NT-1	0.474	0.47	3.89	9.5	11.5	29.1	1081.9	2.0%
NT-2	0.474	0.39	4.29	10.46	15.45	19.41	3317.1	7.7%

**Figure A1:**
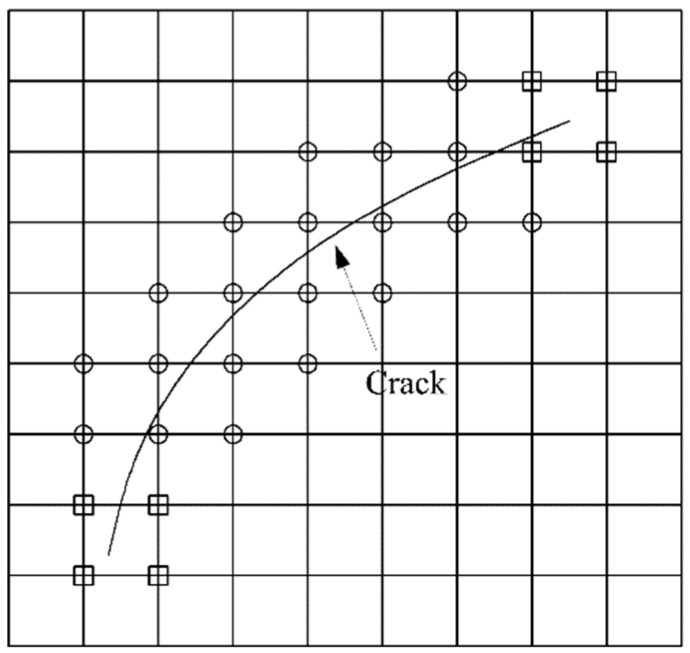
Computational mesh with internal cracks.

**Figure A2:**
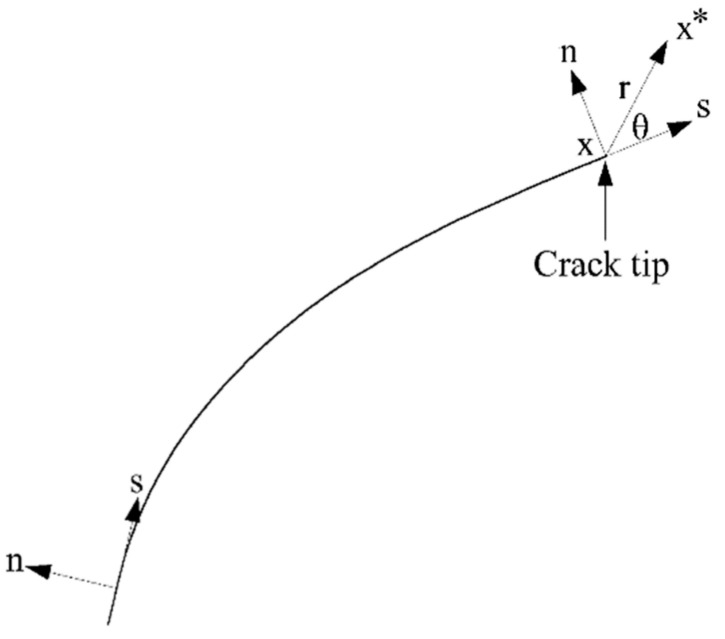
Local coordinates at the crack tip.

**Figure A3:**
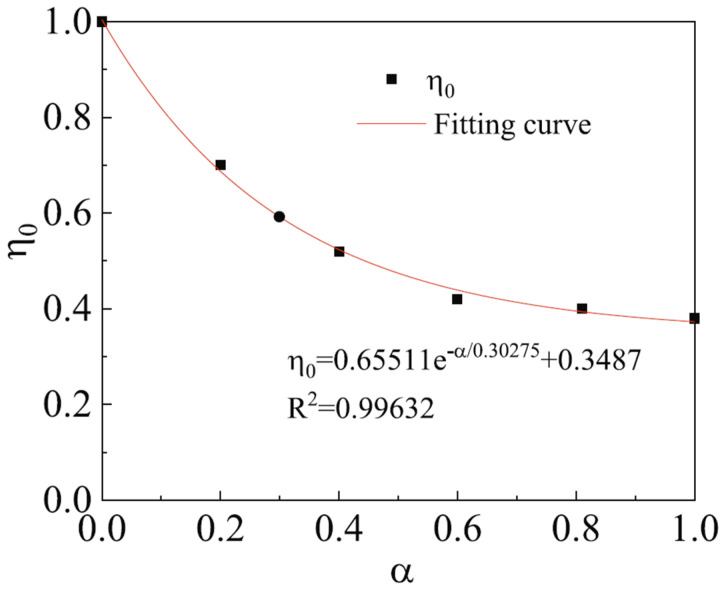
Relationship between *α* and the orientation factor $\eta_{0}$.

**Figure A4:**
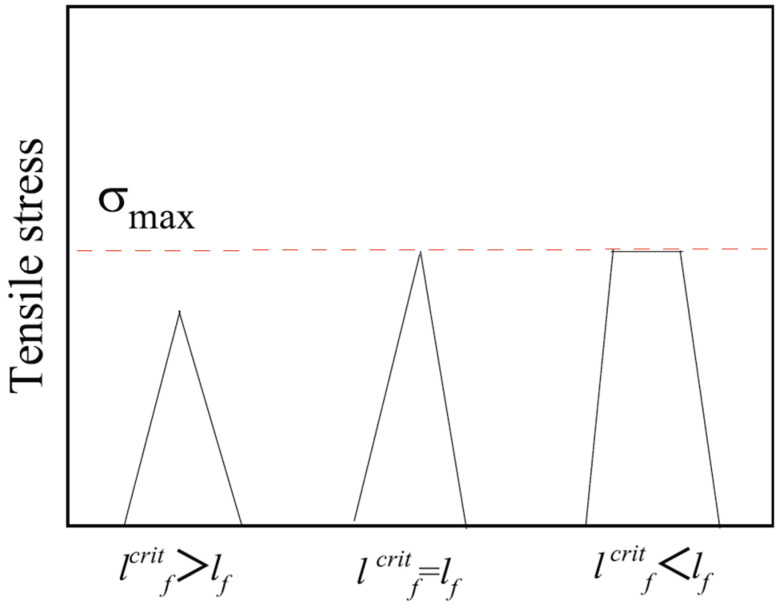
Schematic of fiber tensile stress during failure.

## Data Availability

Data are contained within the article.

## Appendix A

### Appendix A.1. Extended Finite Element Method

Based on the concept of unit decomposition, additional functions that account for the local characteristics of cracks are introduced into the finite element approximation of the displacement. The finite element approximation of the displacement is given by Equation (A1) [39,40,41]:

(A1) $$u=\sum_{i\epsilon \Omega }N_{i}(x)\left[u_{i}+\underset{i\epsilon \Omega_{\Gamma }}{\underbrace{H(x)a_{i}}}+\underset{i\epsilon \Omega_{\Lambda }}{\underbrace{\sum_{l=1}^{4}F_{l}(x)b_{i}^{\left(l\right)}}}\right]$$

where Ω represents the set of all discrete nodes in the mesh; $N_{i}(x)$ is the traditional finite element shape function; $u_{i}$ denotes the traditional nodal degrees of freedom; $\Omega_{\Gamma }$ is the set of nodes for elements with completely tough cracks (indicated by circles in Figure A1), H(x) is the jump function; $a_{i}$ refers to the modified degrees of freedom associated with the jump function; $\Omega_{\Lambda }$ is the set of nodes for elements at the crack tip (indicated by squares in Figure A1); $F_{l}(x)$ is the additional function for the asymptotic displacement field at the crack tip; and $b_{i}^{\left(l\right)}$ represents the modified degrees of freedom associated with the crack tip.

H(x) is the jump function used for elements with tough cracks, and it is defined by Equation (A2) [39,40,41]:

(A2) $$H(x)\left\{\begin{array}{l} +1,\left(x-x^{*}\right)\cdot n>0\\ -1,else \end{array}\right.$$

where x is the point of interest; $x^{*}$ is the closest point on the crack surface to x; and n is the unit normal vector to the crack surface at $x^{*}$.

Since the crack surface does not penetrate through the crack tip elements, the jump function H(x) cannot capture the deformation characteristics at the crack tip. Therefore, it is necessary to introduce additional functions for the crack tip. For isotropic elastic materials, the crack tip additional function can be expressed as follows [39,40,41]:

(A3) $$F_{l}\left(x\right)=\sqrt{r}\left[\sin\left(\frac{\theta }{2}\right)\cos\left(\frac{\theta }{2}\right)\sin\left(\theta \right)\cos\left(\frac{\theta }{2}\right)\sin\left(\theta \right)\sin\left(\frac{\theta }{2}\right)\right]$$

where (r,θ) represents the local coordinate system at the crack tip (as shown in Figure A2).

Once the displacement mode is established, the governing equations can be solved using the principle of virtual work, similar to conventional finite element methods. Assuming the structure undergoes a virtual displacement δu, the virtual work equation is given by Equation (A4) [39,40]:

(A4) $$\int_{\Omega }\sigma \delta \varepsilon d\Omega =\int_{\Omega }f^{b}\delta \varepsilon d\Omega +\int_{\Gamma }f^{t}\delta \varepsilon d\Gamma$$

where $f^{b}$ represents the body forces $and{\;f}^{t}$ denotes the surface forces.

Substituting Equation (A1) into Equation (A4) yields the governing equations:

(A5) Kd=f

In this context, d represents the vector of unknown nodal degrees of freedom, which is expressed as follows [39,40]:

$$d=\left\{u_{i}a_{i}b_{i}^{\left(1\right)}b_{i}^{\left(2\right)}b_{i}^{\left(3\right)}b_{i}^{\left(4\right)}\right\}$$

where K is the global stiffness matrix and f is the external force vector corresponding to the degrees of freedom.

The global stiffness matrix in vector form is expressed as follows:

(A6) $$K=\left[\begin{array}{ccc} K_{uu} & K_{ua} & K_{ub}\\ K_{au} & K_{aa} & K_{ab}\\ K_{bu} & K_{ba} & K_{bb} \end{array}\right]$$

The global stiffness matrix is assembled from the element stiffness matrices. The element stiffness matrix $K_{e}$ is defined as follows:

(A7) $$K_{e}=\int_{\Omega^{e}}{\left(B_{r}\right)}^{T}DB_{s}d\Omega$$

where *r*, *s* = *u*, *a*, *b*; *u*, *a*, *b* correspond to the improved nodal degrees of freedom for conventional elements, through-crack elements, and crack tip elements, respectively; Ω*e* denotes the integration sub-elements, with the crack surface located on the boundaries of these elements; and *D* is the constitutive matrix for isotropic linear elastic materials.

$B_{u}$, $B_{a}$, and $B_{b}$ are matrices of the partial derivatives of the shape functions, computed using the following formulas:

(A8) $$f=\left.\begin{array}{l} B_{u}=\left[\begin{array}{cc} N_{i,x} & 0\\ 0 & N_{i,y}\\ N_{i,y} & N_{i,x} \end{array}\right]\\ B_{a}=\left[\begin{array}{cc} {\left(N_{i}H\right)}_{x} & 0\\ 0 & {\left(N_{i}H\right)}_{y}\\ {\left(N_{i}H\right)}_{y} & {\left(N_{i}H\right)}_{x} \end{array}\right]\\ B_{b}=\left[B_{b1}B_{b2}B_{b3}B_{b4}\right]\\ B_{bl}=\left[\begin{array}{cc} {\left(N_{i}F_{l}\right)}_{x} & 0\\ 0 & {\left(N_{i}F_{l}\right)}_{y}\\ {\left(N_{i}F_{l}\right)}_{y} & {\left(N_{i}F_{l}\right)}_{x} \end{array}\right] \end{array}\right\}$$

where l=1 to 4; $N_{i,x}$ and $N_{i,y}$ represent the partial derivatives of $N_{i}$ with respect to x and y, respectively; ${\left(N_{i}H\right)}_{x}$ and ${\left(N_{i}H\right)}_{y}$ denote the partial derivatives of $\left(N_{i}H\right)$ with respect to x and y, respectively; ${\left(N_{i}F_{l}\right)}_{x}$ and ${\left(N_{i}F_{l}\right)}_{y}$ indicate the partial derivatives of $\left(N_{i}F_{l}\right)$ with respect to x and y, respectively.

The vector f represents the equivalent nodal load vector for the body force b and the external force $\bar{t}$.

(A9) $$f={\left\{f_{u},f_{a},f_{b1},f_{b2},f_{b3},f_{b4}\right\}}^{T}$$

The equivalent nodal load vector is expressed as follows:

(A10) $$\left.\begin{array}{l} f_{u}=\int_{\Gamma_{t}}N_{i}\bar{t}d\Gamma +\int_{\Omega^{e}}N_{i}bd\Omega \\ f_{a}=\int_{\Gamma_{t}}N_{i}H\bar{t}d\Gamma +\int_{\Omega^{e}}N_{i}Hbd\Omega \\ f_{bl}=\int_{\Gamma_{t}}N_{i}F_{l}\bar{t}d\Gamma +\int_{\Omega^{e}}N_{i}F_{l}bd\Omega \end{array}\right\}$$

where l=1 to 4; $N_{i}$ represents the traditional finite element shape functions.

### Appendix A.2. The Application of Composite Material Theory and the Establishment of Specific Parameters

Due to the disorder of basalt fiber bars within the MPC, which reduces the load-carrying efficiency of fibers in the tensile stress direction, a fiber orientation coefficient $\eta_{0}$ is introduced into the calculation model. Additionally, the failure mode of the fibers is closely related to their length; thus, a fiber length coefficient $\eta_{l}$ is also introduced.

For fibers uniformly distributed in the plane and spatial directions, the orientation coefficients $\eta_{0}$ are 0.375 and 0.2, respectively [42]. For a single fiber with a three-dimensional random distribution, the orientation coefficient is influenced by the distance from the fiber center to the specimen edge. When this distance is less than half of the fiber length, boundary effects arise due to the mold’s restriction on the fiber’s free rotation within the MPC. Assuming the distance from the fiber's geometric center to the specimen edge is $\alpha l_{f}$ (which is the fiber length and 0<α<1), the relationship between α and the orientation coefficient $\eta_{0}$ is shown in Figure A3. The average fiber orientation coefficient for all fibers within the specimen is calculated using the formula given in Equation (A11). The calculated average value for the fiber orientation coefficient $\eta_{0}$ in this study is 0.4574.

(A11)
$$\bar{\eta_{0}}=(2\int_{0}^{l_{f}}\eta_{0}dx+\frac{3}{8}(t-2l_{f}))/t$$

After the formation of macro-cracks in the NT and basalt fiber bar reinforced MPC, the load transfers to the fibers until they are either pulled out of or break within the MPC. The calculation formula for the tensile strength of the composite material is given by Equation (A12) [43]:

(A12) $$\sigma_{fc}^{u}=2\eta_{l}\eta_{0}\frac{l_{f}}{d_{f}}\tau V_{f}$$

where $\sigma_{fc}^{u}$ represents the tensile strength of the NT and basalt fiber bar reinforced MPC; $l_{f}$ is the length of the basalt fiber bars; $d_{f}$ is the diameter of the basalt fiber bars; τ denotes the average interfacial bond strength between the basalt fiber bars and the MPC; and $V_{f}$ is the volume fraction of the basalt fiber bars.

The relationship between the bending strength and the tensile strength of the NT and basalt fiber bar reinforced MPC is given by Equation (A13) [44]:

(A13) $$\sigma_{fc}^{b}=2.44\sigma_{fc}^{u}$$

where $\sigma_{fc}^{b}$ represents the bending strength of the NT and basalt fiber bar reinforced MPC, and $\sigma_{fc}^{u}$ denotes the tensile strength of the NT and basalt fiber bar reinforced MPC.

In the non-linear loading phase of the specimen, the failure mode of the internal fibers, whether pull-out or pull-off, is closely related to the fiber length. The relationship between the tensile force on the fibers and their length is shown in Figure A4. From the figure, it can be observed that if the basalt fiber bar length is less than the critical length ($l_{f}<l_{f}^{crit}$), the fibers fail by pull-out when the specimen enters the non-linear phase. When the fiber length equals the critical length ($l_{f}=l_{f}^{crit}$), the fiber failure mode is either fracture at the midpoint or pull-out from the shorter side of the crack. When the basalt fiber bar length exceeds the critical length ($l_{f}>l_{f}^{crit}$), the fiber failure mode is pull-off.

For fibers that fracture at the midpoint, Equation (A14) is established based on force equilibrium.

(A14) $$\pi d_{f}\frac{l_{f}^{crit}}{2}\tau =\frac{\pi }{4}d_{f}^{2}\sigma_{f}^{u}$$

where $l_{f}^{crit}$ denotes the critical length of the fibers. The formula for calculating $l_{f}^{crit}$ is given by Equation (A15), which is derived from Equation (A14):

(A15) $$l_{f}^{crit}=\frac{d_{f}\sigma_{f}^{u}}{2\tau }$$

From Equation (A12), the interfacial bond strength between the MPC and the basalt fiber bars is given by Equation (A16):

(A16) $$\tau =\frac{\sigma_{fc}^{u}d_{f}}{2\eta_{l}\eta_{0}l_{f}V_{f}}$$

When the fiber length $l_{f}$ is less than or equal to the critical length $l_{f}^{crit}$, the fiber length coefficient $\eta_{l}$ is given by Equation (A17):

(A17) $$\eta_{l}=\frac{l_{f}}{2l_{f}^{crit}}.l_{f}\leq l_{f}^{crit}$$

When the fiber length $l_{f}$ is greater than $l_{f}^{crit}$, the fiber length coefficient $\eta_{l}$ is given by Equation (A18):

(A18) $$\eta_{l}=1-\frac{l_{f}^{crit}}{2l_{f}}.l_{f}>l_{f}^{crit}$$

By solving Equation (A15) through Equation (A18) simultaneously, the formula for calculating the critical fiber length $l_{f}^{crit}$ is obtained as follows:

(A19) $$l_{f}^{crit}=\frac{l_{f}\sqrt{\sigma_{f}^{u}\eta_{0}V_{f}}}{\sqrt{2\sigma_{fc}^{b}/2.44}}.l_{f}\leq l_{f}^{crit}$$

(A20) $$l_{f}^{crit}=\frac{2\eta_{0}V_{f}\sigma_{f}^{u}l_{f}}{2\sigma_{fc}^{u}+\eta_{0}V_{f}\sigma_{f}^{u}}.l_{f}>l_{f}^{crit}$$

When the length of the basalt fiber bar satisfies $l_{f}>l_{f}^{crit}$, the average work consumed by a single fiber can be calculated using Equation (A21):

(A21) $$\bar{w_{t}}=\frac{1}{24}\pi d_{f}\tau \frac{{{(l}_{f}^{crit})}^{3}}{l_{f}}$$
